# Delayed Wound Repair in Sepsis Is Associated with Reduced Local Pro-Inflammatory Cytokine Expression

**DOI:** 10.1371/journal.pone.0073992

**Published:** 2013-09-25

**Authors:** Katharina Sommer, Anna Lena Sander, Michael Albig, Roxane Weber, Dirk Henrich, Johannes Frank, Ingo Marzi, Heike Jakob

**Affiliations:** Department of Trauma, Hand and Reconstructive Surgery, Hospital of the Johann Wolfgang Goethe-University, Frankfurt am Main, Germany; The University of Hong Kong, Hong Kong

## Abstract

Sepsis is one of the main causes for morbidity and mortality in hospitalized patients. Moreover, sepsis associated complications involving impaired wound healing are common. Septic patients often require surgical interventions that in-turn may lead to further complications caused by impaired wound healing. We established a mouse model to the study delayed wound healing during sepsis distant to the septic focus point. For this reason cecal ligation and puncture (CLP) was combined with the creation of a superficial wound on the mouse ear. Control animals received the same procedure without CPL. Epithelialization was measured every second day by direct microscopic visualization up to complete closure of the wound. As interplay of TNF-α, TGF-β, matrix metalloproteinases (MMP), and tissue inhibitors of metalloproteinases (TIMP) is important in wound healing in general, TNF-α, TGF-β, MMP7, and TIMP1 were assessed immunohistochemical in samples of wounded ears harvested on days 2, 6, 10 and 16 after wounding. After induction of sepsis, animals showed a significant delay in wound epithelialization from day 2 to 12 compared to control animals. Complete wound healing was attained after mean 12.2± standard deviation (SD) 3.0 days in septic animals compared to 8.7± SD 1.7 days in the control group. Septic animals showed a significant reduction in local pro-inflammatory cytokine level of TNF-α on day 2 and day 6 as well as a reduced expression of TGF-β on day 2 in wounds. A significant lower expression of MMP7 as well as TIMP1 was also observed on day 2 after wounding. The induction of sepsis impairs wound healing distant to the septic focus point. We could demonstrate that expression of important cytokines for wound repair is deregulated after induction of sepsis. Thus restoring normal cytokine response locally in wounds could be a good strategy to enhance wound repair in sepsis.

## Introduction

Sepsis is a state that is marked by a whole body inflammatory response caused by bacterial, fungal or toxic infection. Despite improved therapies sepsis-related complications and their associated morbidity and mortality are increasing in hospitalized patients [1]. Complications involving impaired wound healing such as anastomotic leaks, fascial dehiscence, and infections are common in septic patients [2]. This presents a special problem since patients that suffer from sepsis often require surgical interventions that in-turn lead to further complications caused by the impaired wound healing in sepsis [3]. Despite of this recognized clinical problem, the molecular mechanisms that underlie impaired wound healing in sepsis has not been uncovered yet [4].

The process of wound healing itself is a well-orchestrated local inflammatory reaction to tissue damage. Therefore, it has been proposed that systemic inflammatory response leads to the disruption of this process during sepsis [3], [5]. The tightly regulated expression of cytokines during wound healing is particularly important for the formation of granulation tissue and closure of the wound by epithelialization. This local regulation of cytokines in the wound might be influenced by the systemic elevation of pro-inflammatory cytokines like tumor necrosis factor-alpha (TNF-α) during sepsis leading to a disruption of the inflammatory reaction in the beginning of the wound healing process.

TNF-α can act beneficial or deleterious in a dose dependent manner showing the importance of accurate cytokine regulation in wound healing [6]. Elevation of TNF-α leads to a decrease in the production of granulation tissue whereas low doses of TNF-α promote collagen disposition [7]–[9]. TNF-α also suppresses the function of tumor growth factor-beta (TGF-β) as it counteracts the production of extracellular matrix (ECM) that is enhanced by the later cytokine [10]. TGF-β induces production of ECM by stimulating collagen production [10]. This is partly achieved due to inhibition of matrix metalloproteinases (MMPs) by up-regulation of tissue inhibitors of metalloproteinases (TIMPs) that negatively regulate MMP function [11]. In contrast to this function of TGF-β, TNF-α down-regulates TIMPs and enhances the expression of MMPs supporting the activity of these MMPs [6]. MMPs are important for restoration of normal tissue architecture and take part in activation and degradation of cytokines, angiogenesis, epithelialization and scar formation [12]. MMPs have multiple roles in the regulation of inflammatory response after tissue injury, and disruption of MMP expression leads to an altered immune response assigning MMPs a major function in the regulation of inflammation in local wounds [9].

As these cytokines and MMPs are important for normal wound healing they might be disturbed in the state of sepsis. In this study we created a mouse model where we combined the cecal ligation and puncture model for induction of sepsis with the creation of a wound that is locally separated to the focus of sepsis. We analyzed local cytokine levels throughout wound healing in sepsis in comparison to cytokine levels in wounds of non-septic animals in order to investigate whether systemic inflammation influences the local wound healing process.

## Materials and Methods

To analyze wound repair during sepsis, a model of cecal ligation and puncture was combined with the simultaneous creation of a wound on the dorsal side of the mouse ear [13]. Subsequently the inflammatory reaction was defined by measurement of IL-6 and TNF-α in the serum on the day of sacrifice. Epithelialization of the wounds was measured *in vivo* in animals. For analysis of cytokine expressions and MMPs in wounds, animals were sacrificed on day 2, 6, 10, and 16 after wound creation.

### Animals

All animal experiments were performed in accordance with ethic guidelines of German law and approved by the Regierungspräsidium Darmstadt (Ethic Approval no. V54-19c20/15-F3 7 K 2244).

Female hairless SKH-1 mice (weight 20–30 g; age 6–8 weeks) were obtained from Charles River Laboratories (Sulzfeld, Germany). Animals were housed in separate cages at 24°C with light intervals of 12 h/day in airflow regulated rooms, and fed a balanced rodent diet with water *ad libitum*. For all surgical interventions the animals were anesthetized with intraperitoneal (i.p.) injection of 100 µl solution containing 2.215 mg of ketamine and 0.175 mg of xylazine hydrochloride. For all wound measurements animals were anesthetized the same as for surgery. For post-operative analgesia the animals received buprenorphin (1g/kg body weight) subcutaneously twice daily. At the end of the experiments animals were euthanized by cervical dislocation.

### Induction of sepsis

Polymicrobial sepsis was induced by CLP as described previously [14]. Through a midline laparotomy the cecum was identified and carefully retrieved from the abdominal cavity, ligated 7 mm above its blunt end and punctured with a 29-gauge needle. A small amount of cecal content was carefully squeezed out through the puncture wound, the caecum was returned to the abdominal cavity. Animals were then given 1 ml 0.9% NaCl solution i.p. for fluid resuscitation and their abdominal cavity was closed in two layers (5–0, nylon). Control animals received the same laparotomy and exposition of the cecum without ligation and puncture of the cecum.

### Analysis of the septic inflammatory reaction

Serum IL-6 and TNF-α were measured in serum as markers of systemic inflammation by BD™ *Cytometric Bead Array*® (CBA, BD Biosciences, San Diego, USA) and flow cytometry according to manufacturer's instructions. Briefly, heparinized blood from sacrificed animals was centrifuged at 2000 g to obtain the serum samples. 50 µl of diluted serum samples (1∶10 in PBS) and serially diluted standards were incubated with 50 µl of mixed capture bead suspension (IL-6 and TNF-α) for 1 hour at room temperature.

After incubation, 50 µl of Phycoerythrin-linked detection reagent was added. After 1 hour samples were washed once by adding 1 ml of wash buffer and subsequent centrifugation. For analysis by flow cytometry, beads were resuspended in 300 µl wash buffer. Results were quantified using *FACP Array*® software (Biosciences, San Diego, USA).

Because the spleen as the largest lymphatic organ in the body is also important for the inflammatory response during sepsis, spleens were weighed on day of sacrifice to evaluate hypertrophy as a sign for prolonged inflammation.

### Wound Creation

Wounds were created on the dorsum of both ears immediately after the sepsis induction procedure. A circular punch (diameter 2.25 mm) was used to incise a full thickness layer of ear skin down to the underlying cartilage, as described previously to attain a standard size of the wounds [13]. After the punch incision was made, a full thickness layer of skin was carefully dissected away from the underlying cartilage, the wound was covered with a sheet of 2.5% methylcellulose in PBS and the entire ear was covered with a bioadhesive dressing (Opsite; Smith and Nephew Medical Ltd., Tuttlingen, Germany).

### Wound epithelialization and wound closure measurements

As previously described the wound model on the mouse ear allows direct measurement of epithelialization of the wound because wound contraction that occurs normally in mice is impeded as the skin of the ear is directly attached to the underlying cartilage. Thus epithelialization could directly be measured in living animals.

On the day of wounding (day 0) and subsequently every second day thereafter, wound epithelialization was measured by direct visualization and quantification using *in vivo* microscopy and computerized planimetry. Measurements were performed by placing the outstretched ear of anesthetized animals on an acrylic glass platform on the stage of an intra-vital microscope (Carl Zeiss, Oberkochen, Germany). The microscope image was captured with a low light camera (DXC-390P, 3CCD color video camera; Sony, Tokyo, Japan) and transferred through a digital converter (ADVC-100; Canopus, Ruppach-Goldhausen, Germany). Digitalized images were analyzed using the ImageJ software (http://rsb.info.nih.gov/ij/download.html) by tracing the wound margin and calculating the area. The rate of wound-closure was expressed as the ratio of the wounded area at each time point divided by the original wound area at time 0.

Wound closure was determined as the day of complete epithelialization of the wound. Analysis was performed by an independent investigator.

### Evaluation of inflammatory reaction in wounds

Wounded ears from sacrificed animals were embedded in TissueTek (Sakura Finetek Europe, Zoeterwoude, The Netherlands) on day 2, 6, 10, and 16 after surgery and stored at –80°C. For immunohistochemical staining, wounds were cut to sections of 5 µm thickness.

For analysis, the sections were submerged in acetone (–20°C, 10 min) followed by 10 min 0.1% hydrogen peroxidase treatment. Sections were stained with primary antibodies directed against TNF-α (Abcam, Cambridge, UK) and TGF-β (Abcam, Cambridge, UK) for wound cytokine expression, and MMP7 (Abbiotec, San Diego, USA) as well as TIMP1 (Abbiotec, San Diego, USA) for 1 h at RT. All primary antibodies were purchased from Abcam (Cambridge, UK). Primary antibodies were detected by HRP-AEC (Abcam, Cambridge, UK) staining according to the guidelines of the manufacturer. Sections were counterstained with hematoxylin and viewed at 100× magnification (Axio Observer; Carl Zeiss, Oberkochen, Germany). The microscope image was captured with a low light camera (AxioCam; Carl Zeiss, Oberkochen, Germany) and digitalized. Photographic images were analyzed using the ImageJ software. The staining of each section was standardized to the area scanned. The analysis was performed by an independent investigator.

### Statistical Analysis

Data are presented as the mean ± SD or box blot of the median. Statistical evaluation was performed with non-parametric Wilcoxon-Mann-Whitney-U-Test. Values of p<0.05 were considered statistically significant. The number of samples examined is indicated by n.

## Results

### Induction of sepsis in animals

**Figure 1:**
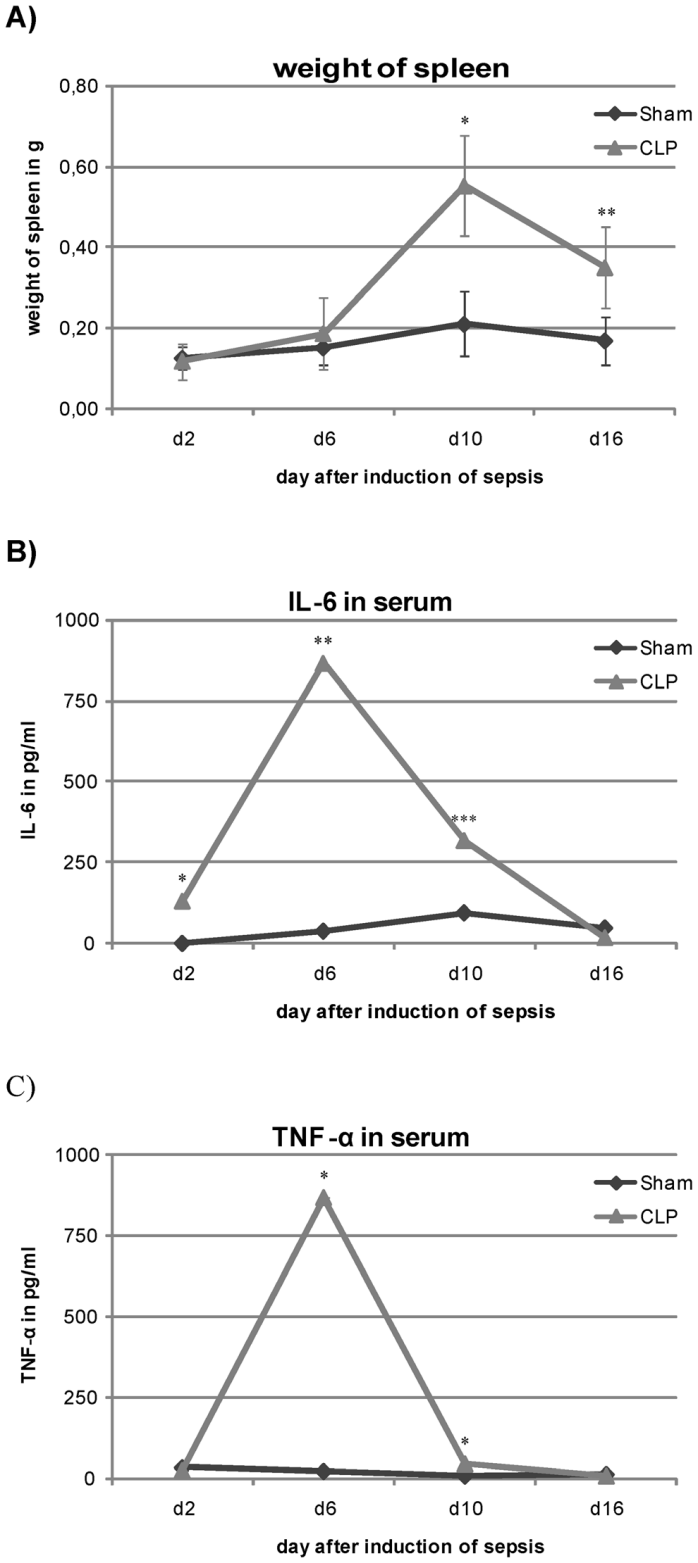
Measurement of systemic inflammatory reaction in mice after CLP compared control. A. Weight of spleen on day of sacrifice. Septic animals show an increase of weight of spleen on day 10 and 16 after induction of sepsis compared to Sham operated animals (Data is shown as mean ± SD, *n* = 8). B. Serum level of IL-6 on day of sacrifice. Mice that underwent CLP procedure show a significant increase in IL-6 serum level on day 2, 6 and 10 after surgery compared to control animals (Data is shown as mean ± SD, *n* = 7). C. Serum level of TNF-α. In correlation to IL-6 level in the blood septic mice show also an augmented level of TNF-α on day 6 and 10 after CLP (Data is shown as mean ± SD, *n* = 7). * p<0,001, ** p<0,01, *** p<0,05.

To verify sepsis after CLP procedure we measured IL-6 and TNF-α level in the serum as a marker for the systemic inflammation in septic and non-septic mice on the day of sacrifice. For prolonged inflammatory reaction weight of spleen was also evaluated. Septic animals showed a significantly increased weight of spleen on day 10 with 0.55 g and 16 with 0.35 g after induction of sepsis compared to non-septic mice that had a normal weight of spleen with 0.21 g on day 10 and 0.17 g on day 16 (p<0,001 for day 10 and p<0.01 for day 16; Fig. 1A). Animals that underwent CLP procedure also showed significantly higher serum level of TNF-α on day 6 with 867.5 pg/ml and 10 with 45.3 pg/ml compared to control mice with 23.4 pg/ml and 9.1 pg/ml respectively (p<0.001 for both; Fig. 1B). IL-6 level was also elevated on day 2 with 130.5 pg/ml, day 6 with 867.5 pg/ml and day 10 with 317.8 pg/ml compared to control with 1.9 pg/ml on day 2, 38.8 pg/ml on day 6, and 93.7 pg/ml on day 10 (p<0.001 for day 2, p<0.01 for day 6 and p<0.05 for day 10; Fig. 1C).

### Re-epithelialization and wound closure

**Figure 2:**
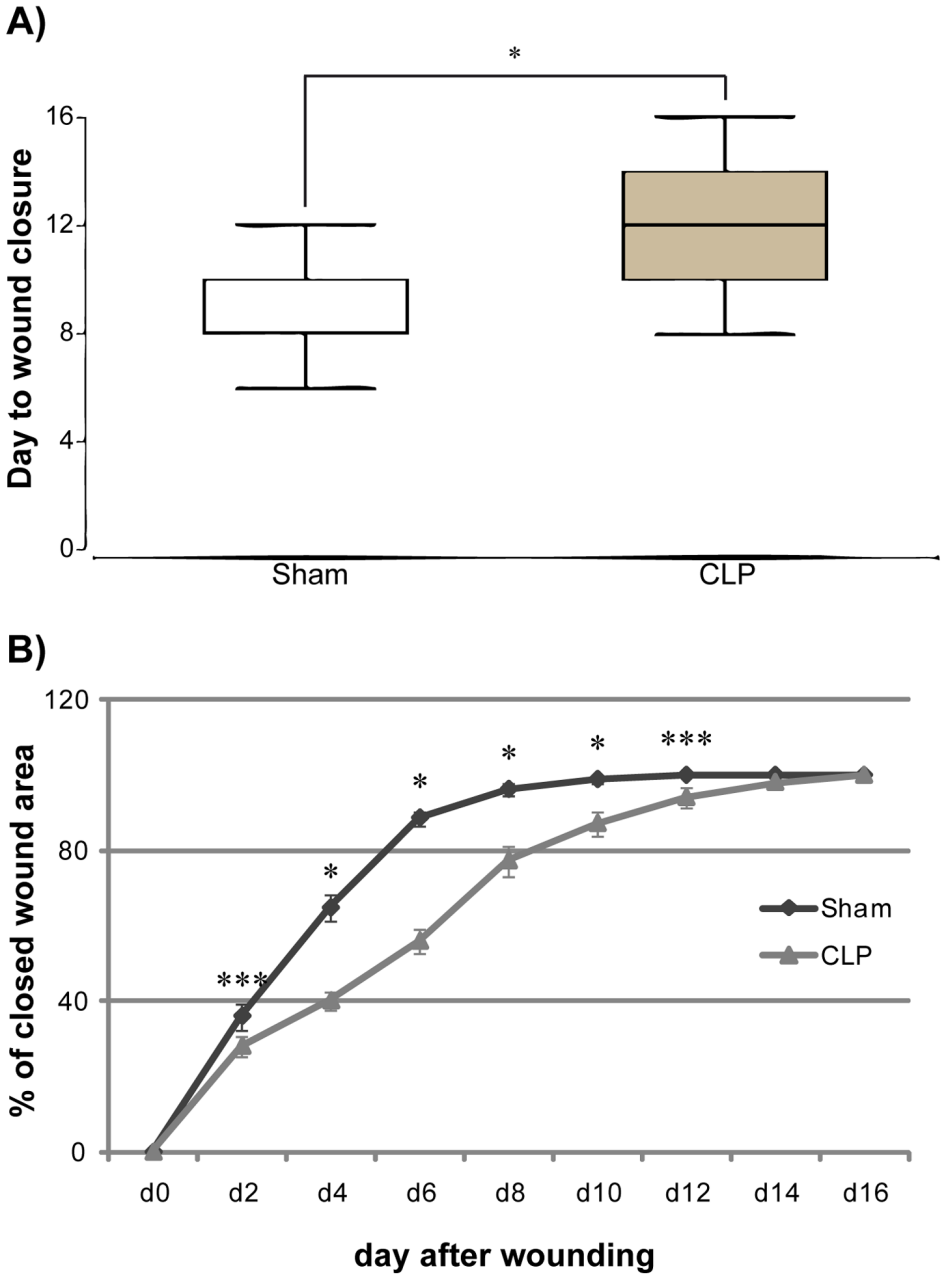
Delay of wound closure and epithelialization in septic animals. A. Day of wound closure. Animals that underwent CLP procedure exhibit a highly significant delay in day of wound closure (Data is shown as box plots of the median, *n* = 10). B. Percentage of closed wound area on day 0–16 (measured every second day). Septic mice have a highly significant delay in epithelialization as there is a decrease in percentage of closed wound area from day 2 to day 12 after wounding. (Data is shown as mean ± SD, *n* = 10). * p<0,001, *** p<0,05.

After confirmation of high inflammatory response in septic animals, we evaluated re-epithelialization and wound closure in septic and non-septic animals as this is uniquely possible in the used model due to missing wound contraction of wounds on the mouse ear as the skin of the ear is adhering to the underlying cartilage. Animals that received CLP procedure showed a significant delay regarding the day of wound closure compared to normal animals as wound closure occurred in non-septic animals occurred on day 8.7 (mean ±1,7 standard deviation, SD) compared to septic animals that showed fully closed wounds on day 12.2 (mean ±3,0 SD, p<0.001; Fig. 2A). The epithelialization of the wounds was also significantly delayed throughout the whole wound healing as septic animals displayed a significantly larger wound area from day 2 to day 12 compared to control (p<0.05 for day 2 and 12, p<0.001 for day 4, 6 and 10; Fig. 2B).

### Cytokine expression in wounds

As initial inflammatory response is a key step to wound repair, we analyzed cytokine levels throughout wound healing monitoring the inflammatory response after wounding in septic and non-septic animals.

TNF-α is one of the most important pro-inflammatory cytokines that is highly elevated during inflammation in sepsis. As serum levels of TNF-α were increased, we were interested to know whether there was also an increase of this cytokine in the wound side when comparing septic to non-septic animals.

**Figure 3:**
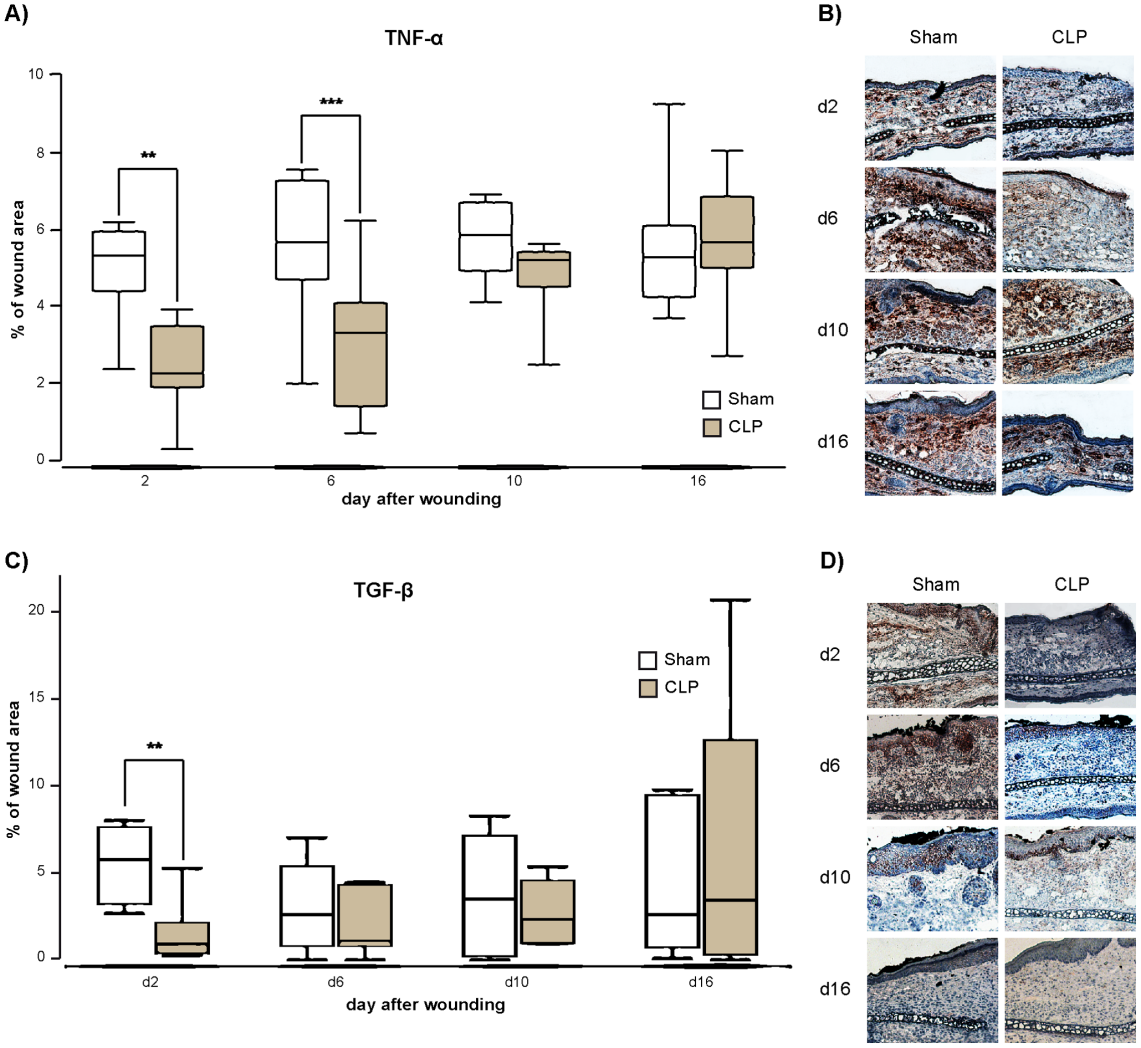
Expression of TNF-α and TGF-β in wounds. A. Percentage of TNFα-positive area on days 2, 6, 10 and 16 after wounding. Septic animals express significant less TNF-α compared to control animals on day 2 and 6 after wounding. (Data are expressed as box plots of the median, *n* = 8). B. Representative pictures of TNF-α staining at the wound margin captured under 100x magnification of sham and CLP animals on day 2, 6, 10 and 16 after staining for TNF-α. Wound margin is located on the right side. C. Percentage of TGF-β-positive area in epidermis on days 2, 6, 10 and 16. Mice that underwent CLP procedure show a reduced level of TGF-β on day 2. (Data are expressed as box plots of the median, *n* = 7). D. Representative pictures of TGF-β staining at the wound margin captured under 100x magnification of sham and CLP animals on day 2, 6, 10 and 16 after staining for TGF-β. Wound margin is located on the right side. ** p<0,01; *** p<0,05.

Immunhistochemical analysis showed a significant reduction of TNF-α on day 2 and 6 in wounds of septic animals (p<0.01 on day 2 and p<0.05 on day 6; Fig. 3A). On day 10 there also was a diminished level of this cytokine in wounds of mice that received CLP procedure, though this difference was not significant (p = 0.06). Equal level of TNF-α to the non-septic animals were obtained only on day 16 after wounding.

As TGF-β is also important to the repairing process and partly counteracts TNF-α in wound healing, we also analyzed the expression of TGF-β in the epidermal layer. We found that on day 2 there was significant decrease of TGF-β expression in wounds of septic animals compared to non-septic mice (p<0.01, Fig. 3C). There was also a tendency of less TGF-β on day 6 in the wounds of mice that underwent CLP procedure though this was not significant in our experiments (p = 0.53, Fig 3C).

### Expression of matrix metalloproteinase and tissue inhibitor of matrix metalloproteinase

As the regulation of MMPs and their inhibitors are important for re-epithelialization and tissue repair and take part in regulating inflammatory response in wounds, we looked at the expression of these proteins in wounds of septic and non-septic animals.

**Figure 4:**
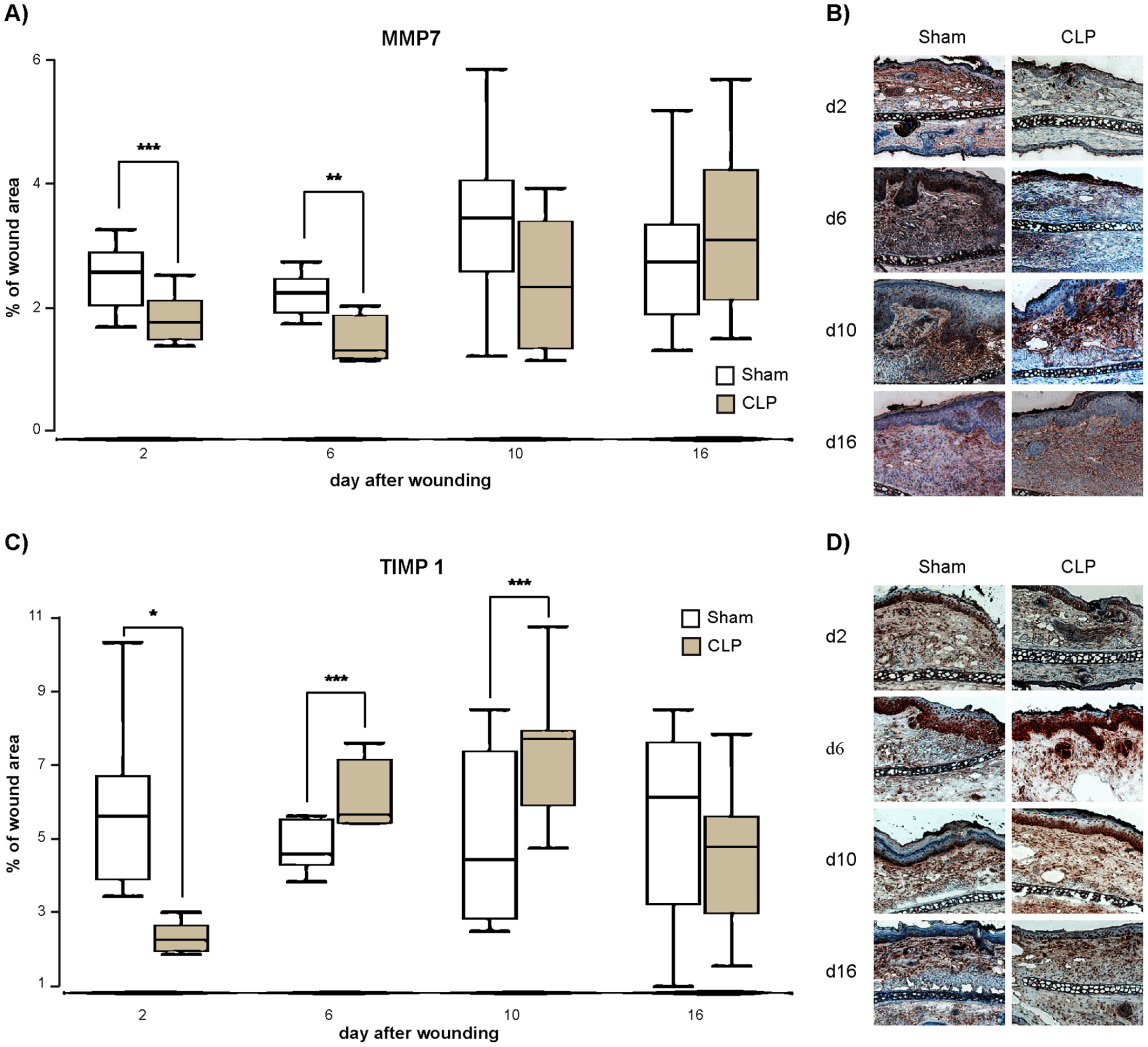
Expression of MMP7 and TIMP1 in wounds. A. Percentage of MMP7-positive area on days 2, 6, 10 and 16 after wounding. Septic animals show a significant decrease in MMP7 expression on day 2 and 6 compared to control mice. B. Representative pictures of MMP7 staining at the wound margin captured under 100x magnification of sham and CLP animals on day 2, 6, 10 and 16 after staining for MMP7. Wound margin is located on the right side. C. Percentage of TIMP1-positive area on days 2, 6, 10 and 16. TIMP1 was significantly decreased on day 2 after wounding after CLP. In the later phases of wound repair a significant increase of TIMP1 was observed in septic mice compared to control animals. D. Representative pictures of TIMP1 staining at the wound margin captured under 100x magnification of sham and CLP animals on day 2, 6, 10 and 16 after staining for TIMP1. Wound margin is located on the right side. (Data are expressed as box plots of the median *n* = 8). * p<0,001, ** p<0,01, * p<0,05.

On day 2 and day 6 after wounding a significant lower expression of MMP7 was observed in wounds of septic animals compared to controls (p<0.001 on day 2 and p<0.01 on day 6, Fig. 4A). In later phases of wound healing on day 10 a decrease in MMP7 expression in mice that underwent CLP procedure could still be noticed thought this difference was not significant.

On day 2, TIMP-1 expression was significantly lower in wounds of septic animals compared to control (p<0.001; Fig. 4C). Septic mice showed a significantly higher level of TIMP-1 in wounds than their non-septic counterparts in the later phases of wound healing (p<0,05, Fig. 4C).

## Discussion

Impaired wound healing is a common problem in septic patients. While sepsis leads to a systemic hyperinflammatory response, the role of local inflammatory response in wounds during sepsis has not been elucidated clearly [3].

In this study, we established an animal model of wound healing in sepsis combining CLP with the creation of a wound that is locally separated from the focus of the sepsis. We could affirm that systemic sepsis causes a delay in wound healing in a remote wound without contact to the source of sepsis as it has already been shown in the work of other groups [3], [4], [15], [16]. In general, wound healing in mice is a combined process of contraction and epithelialization, which distinguishes the wound healing in mice from human wound healing, as in men contraction does not contributes to wound healing in such an extent [17]. In previous studies it has been shown that the process of wound healing located at mice ears as used in the current study, does not contain contraction to a significant amount [13]. Therefore, it can be assumed that wound healing investigated in the current study is mostly due to epithelialization and not contraction, thus mimicking the process more accurately to wound healing in humans.

During sepsis local wound healing might be disturbed by a deregulation of cytokine expression in wounds caused by systemic inflammation. Indeed, in the current study a decrease in local TNF-α and TGF-β expression was observed in the wounds. In contrast, systemic serum levels of pro-inflammatory cytokines of TNF-α and IL-6 were elevated during the first days after induction of sepsis and the creation of the wound verifying the septic state of the mice.

TNF-α is an important cytokine for wound healing. It has been shown that local elevation of TNF-α expression is able to enhance wound repair while depletion of TNF-α has the opposite effect [18]. TNF-α at the wound site directly or indirectly, stimulates fibroblast proliferation, promotes re-epithelialization and neo-vascularization, and enhances wound breaking strength. This supports our findings that decreased TNF-α in the wound of septic animals might be a cause for impaired wound healing [18], [19].

On the other hand Maish et al. found that inhibition of TNF-α by a TNF-α binding protein improves the altered wound healing process in colonic anastomosis that is impaired by sepsis encouraging the hypothesis that TNF-α is involved in the delay of wound healing. This also illustrates the controversial role of this cytokine during wound healing [6], [20]. These differences could be explained by diverse experimental designs. Maish et al. aimed to investigate anastomotic leakage in sepsis and therefore used a model where the wound is close to the septic focus [20]. In contrast, we examined wounds that were locally separated from the focus of sepsis which could explain the different findings. Moreover in the mentioned study TNF-α binding protein was administered systemically which might have had an influence on systemic inflammatory response [20]. In contrast we compared the expression of local TNF-α in wound of septic to non-septic animals without pharmacological intervention.

Our observation that sepsis induces a reduced level in local TNF-α production is further supported by the notion that invading neutrophils and macrophages in wounds are depleted in septic animals whereas the neutrophil counts in peripheral blood are elevated [4]. As these immune cells are a main source for pro-inflammatory cytokines, a local decrease in the number of neutrophils and macrophages may have a negative effect on local TNF-α expression in the wounds of septic animals as found in the current analysis [21]. This should be elucidated in further studies.

We also observed a reduced expression of TGF-β in the wounds of septic animals. TGF-β1 is an essential cytokine for initiation of inflammation as well as the formation of granulation tissue [22]. It is mainly produced by platelets, monocytes and fibroblasts in the wound thus reduced attraction of monocytes to the wound side as it is found during sepsis could be a cause for the decreased expression of this cytokine in wounds of septic mice [23]. TGF-β also takes part in attracting neutrophils, macrophages and fibroblasts to the wound side [10]. Thus reduced level of TGF-β could contribute to impaired homing of these cells to the wound during sepsis. Moreover, TGF-β1 is involved in up-regulating MMPs that are important for cell migration during wound repair leading to a delay in re-epithelialization [10]. It also stimulates wound contraction by induction of α-smooth muscle actin in fibroblasts [22]. Furthermore, TGF-β deficient mice show an impaired wound repair with a deceleration in the re-epithelialization process. In our study we also noticed a deceleration in wound closure that might partly be explanted by a decreased expression of TGF-β and MMPs.

Finally, in our model a deregulation in MMPs and their inhibitors could be noticed. MMPs and their inhibitors are important local regulators for inflammation [12]. They take part in activation and degradation of cytokines and are important for the restoration of normal tissue architecture [12]. These enzymes are involved in re-epithelialization and angiogenesis in the wound. [12]. In the current study, we found a dysregulation in expression of local MMPs and their inhibitors. Wounds of septic animals initially exhibited a decrease in MMP7 as well as TIMP1 expression. During the later stages of wound healing, septic animals displayed an increased level of TIMP1 although MMP levels equaled that of control animals.

TGF-β and TNF-α are regulated by MMPs and they are among the most important enzymes for tissue remodeling and are essential to cleavage of extracellular matrix proteins, modifying cell-cell and cell-matrix interaction, and activation of growth factors thereby supporting tissue remodeling, neovascularization and migration of cells [24]. MMP-7 also takes part in releasing TNF-α from macrophages and contributes to establish a local chemokine gradient during the initial phase of wound healing [24]. Thus the deregulation of proteinases in wound healing during sepsis might be one mechanism that takes part in inadequate inflammatory response after wounding and leads to impaired healing.

Greenhalgh et al. proposed in their study on wound repair during sepsis that the nutritional depletion that is present in the first days after induction sepsis could be a cause for the deterioration in wound healing in septic mice [15]. This conclusion was based on findings in non-septic mice, that were pair fed to septic animals and consecutively exhibited a similar delay in wound repair and wound breaking strength as their septic counterparts [15]. Nevertheless, this finding could not be verified within a later study where septic mice showed a delay in wound repair whereas the non-septic pair fed counterparts displayed normal wound healing [16]. However, the catabolic metabolism that occurs during sepsis despite adequate nutritional support might play a role in the delay of wound healing though catabolism does not seem to be the only reason for the impairment [25].

Albeit this possible correlation between malnutrition and impaired wound repair, there is also clinical evidence for the delay in wound healing due to sepsis. In a clinical study, septic patients showed a decelerated wound repair after induction of burn wounds compared to control patients indicating that malnutrition might not be the only reason for impaired healing since patients on intensive care units receive an optimal nutritional supply [3].

In conclusion, we found an impaired wound healing in septic animals. Impaired wound healing was correlated with a decreased expression of cytokines that are essential for the wound healing process. Moreover reduced level of MMP7 with an altered TIMP1 expression was found in the wounds of septic animals. Thus, systemic hyperinflammation seems to interfere with the regular local inflammatory healing response. Further studies will be needed to clarify whether this deregulation in cytokines is the major cause for the deterioration in healing during sepsis and if normalizing cytokine level could be a means for regaining normal wound repair.
